# Substantial Variations in the Optical Absorption and Reflectivity of Graphene When the Concentrations of Vacancies and Doping with Fluorine, Nitrogen, and Oxygen Change

**DOI:** 10.3390/ijms22136832

**Published:** 2021-06-25

**Authors:** Ali Fransuani Jiménez-González, Juan Manuel Ramírez-de-Arellano, Luis Fernando Magaña

**Affiliations:** 1 Instituto de Física, Universidad Nacional Autónoma de México, Apartado Postal 20-364, Ciudad de México 01000, Mexico; alifransuanijg@gmail.com; 2 Tecnologico de Monterrey, Escuela de Ingeniería y Ciencias, Av. Eugenio Garza Sada 2501, Monterrey 64849, Mexico; radear82@gmail.com

**Keywords:** graphene, optical properties, nitrogen, oxygen, fluorine, vacancies

## Abstract

We performed ab initio numerical simulations with the density functional theory to investigate the variations in the band structure, optical absorption, and the reflectivity of vacancy-graphene doped with nitrogen, oxygen, and fluorine for different densities. We considered the density values 0.78%, 1.02%, 1.39%, 2.00%, 3.12%, 5.55%, and 12.5% for the vacancies and doping. In the infrared and visible ranges for all cases, vacancies included, there is a substantial increment in the absorption and reflectivity concerning graphene. The most significant changes are for fluorine and oxygen at a concentration of 12.5%.

## 1. Introduction

The fact that semiconductors have electrical and optical properties tunable by changes in the band structure is one of continuing importance and interest. Recent research on graphene and other 2D materials [1] has focused on bandgap opening to unlock applications such as electrodes [2]. Other studies have achieved a bandgap corresponding to visible light (1.6–1.7 eV), essential for applications in photocatalysis [3,4].

One of the main ways of tuning the graphene’s bandstructure is doping it using different atoms. Using nitrogen has shown to be relevant for energy, sensor development, and environmental applications [5,6]. Oxygen is another studied dopant candidate. The adsorption of oxygen atoms at a single graphene site has yielded an opening of the bandgap of about 0.6 eV [7]. In this case, the research lines focus on several applications. Among these, the sensors [8,9] and water desalination applications [10]. Another investigated aspect is the oxygen evolution reaction [11], or the oxygen reduction reaction [12]. Similarly, we have the tailoring of optical and electrical properties [13,14].

Combining nitrogen and fluorine to dope graphene resulted in good electrochemical performances when tested as the anode for lithium-ion batteries [15]. However, doping with fluorine alone also affects the electrical properties [16] and chemical stability of graphene [17]. Fluorine-doped graphene has shown the potential to be an efficient, cost-effective, and durable catalyst for its use in the fuel cell industry [18].

Other authors’ studies focused on tuning the band structure via the symmetry of the dopant atoms to the graphene surface and modifying the graphene positions concerning the substrate [19,20]. Studies involving fluorine, nitrogen, and oxygen have helped compare the different resulting properties obtained for each of them, although they have focused on a single value dopant concentration [21,22].

This work explores how graphene’s band structure and its optical properties change by varying graphene vacancy density or dopant concentration. The band structure, optical absorption, and reflectivity variations are useful tools for electronic and optical applications and detecting dopants and defects in the surface. We considered fluorine, nitrogen, and oxygen as dopants in the graphene vacancies. The concentration values we took were 0.78%, 1.02%, 1.39%, 2.00%, 3.12%, 5.55%, and 12.5%. We performed first-principles numerical simulations.

## 2. Results

### 2.1. Optimized Structures

**Figure 1:**
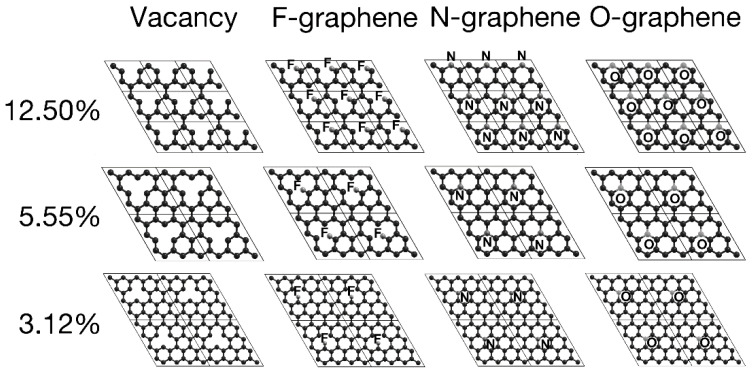
Top view of the XY plane for graphene vacancy/dopant at: 12.5% top, 5.55% middle, and 3.12% bottom. We mark the boundaries of the unit cells with lines.

**Figure 2:**
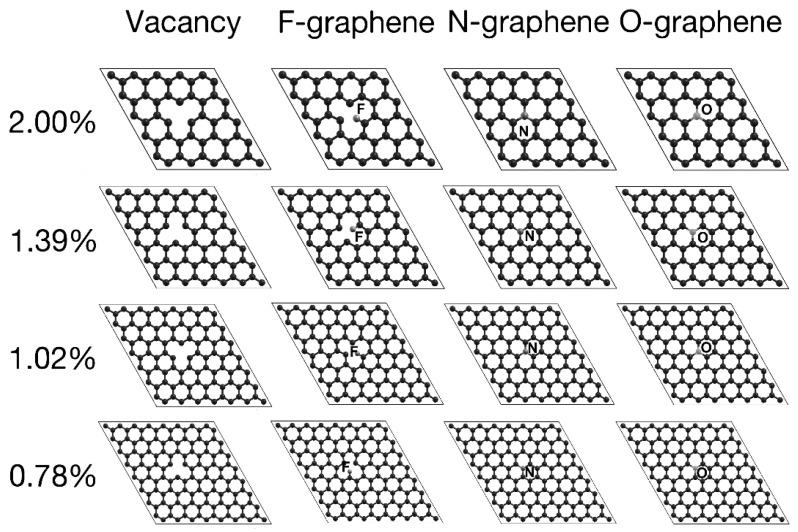
Top view of the XY plane for graphene with vacancy/dopant at: 2%, 1.39%, 1.02%, and 0.78%. We mark the boundaries of the unit cells with lines.

We considered seven configurations in terms of the dopant density, performing structural relaxations for each. Table 1 shows the number of atoms per unit cell mentioned above, their corresponding doping percentage, and the corresponding adsorption energies with each of the three dopants separately. Figure 1 and Figure 2 show the unit cells we used for the different vacancy-dopant ratios.

The graphene surface’s most prominent deformation occurs for the F dopant–this case is as F-graphene. The N dopant–labeled as N-graphene–incorporates smoothly into the lattice without deforming it, apart from a slight displacement of 0.1 Å over the graphene plane, observed only at dopant densities of 5.55% and 12.5%. For the case of O-graphene, while increasing the dopant density, its distance from the surface slightly decreased, remaining approximately 0.5 Å from the graphene sheet.

Adding dopants of similar size to carbon could be incorporated into the lattice while preserving the structure, as shown when nitrogen and oxygen adsorbed in the graphene’s plane. Whereas fluorine, being more prominent and with one missing electron to fill its shell, tends to bond with only one carbon. Furthermore, this bond deforms the graphene lattice, creating a bulge in the system.

All oxygen and nitrogen percentages bounded with the three-carbon surrounding the vacancy, with an average bound size of 1.53 and 1.42 Å, respectively. O-graphene has a bond length of 0.11 Å greater than pristine graphene, bringing oxygen slightly out of the graphene’s plane. In comparison, F-graphene is bound to one carbon, with an average bound distance of 1.38 Å.

The distances between carbons remain almost constant regardless of the dopant percentages. The separation between the first neighbors is an average of 1.3871 Å in the vacancies cases. F-graphene maintains an average distance of 1.4021 Å. O-graphene keeps an average spacing of 1.3889 Å (same as with vacancy), while in the case of nitrogen, the average spacing is 1.4151 Å.

**Figure 3:**
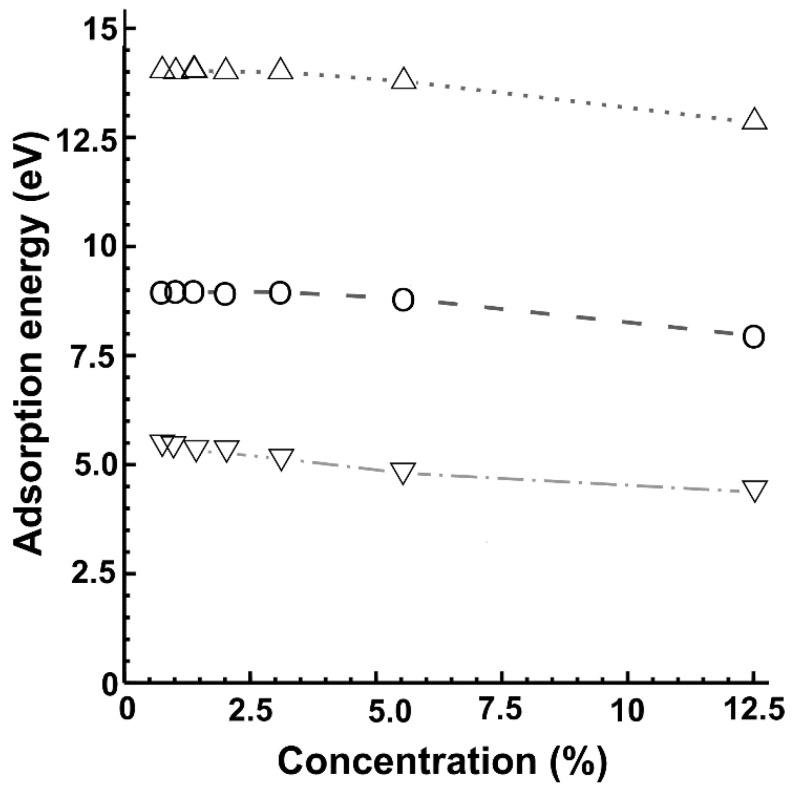
We present the absorption energies for each of the three dopants and vacancy as a function of the dopant density. For nitrogen: △; for oxygen: **Ο**; for fluorine: **▽**.

Figure 3 shows the variation of the adsorption energy with the dopant density. The absorption energies decrease almost linearly as the percentage increased. Nitrogen presented the highest adsorption energy, and fluorine the lowest. All the values correspond to intense interactions and stable adsorptions.

### 2.2. Projected Density of States (PDOS)

**Figure 4:**
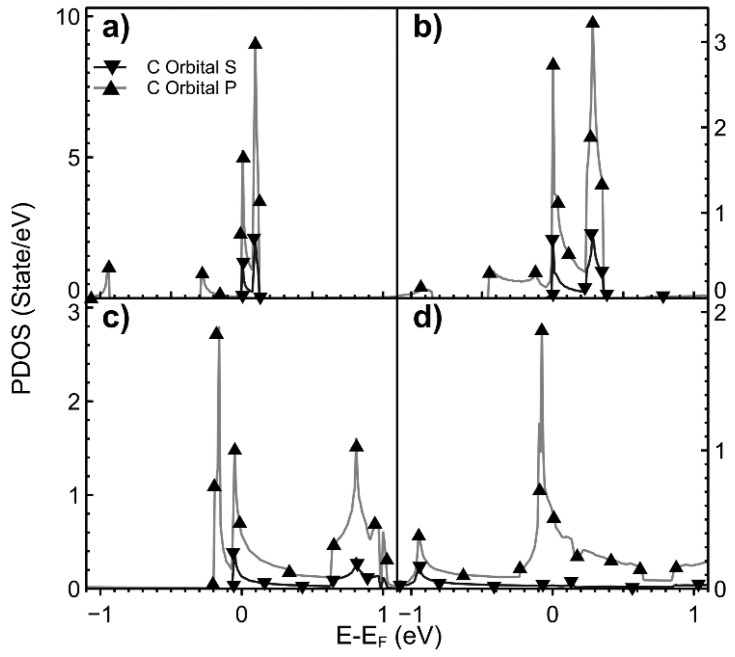
We show the PDOS for different values of vacancy densities. (**a**) 2.00%; (**b**) 3.12%; (**c**) 5.55%; (**d**) 12.5%. Orbitals *s* (▼) and *p* (▲) for graphene with a vacancy.

In Figure 4, we show the variation of the PDOS as the vacancy density changes. The *s* and *p* states’ hybridization for the lowest vacancy density is mainly in the Fermi energy neighborhood. As this density increases, the *s* and *p* hybridization spreads beyond the Fermi energy.

**Figure 5:**
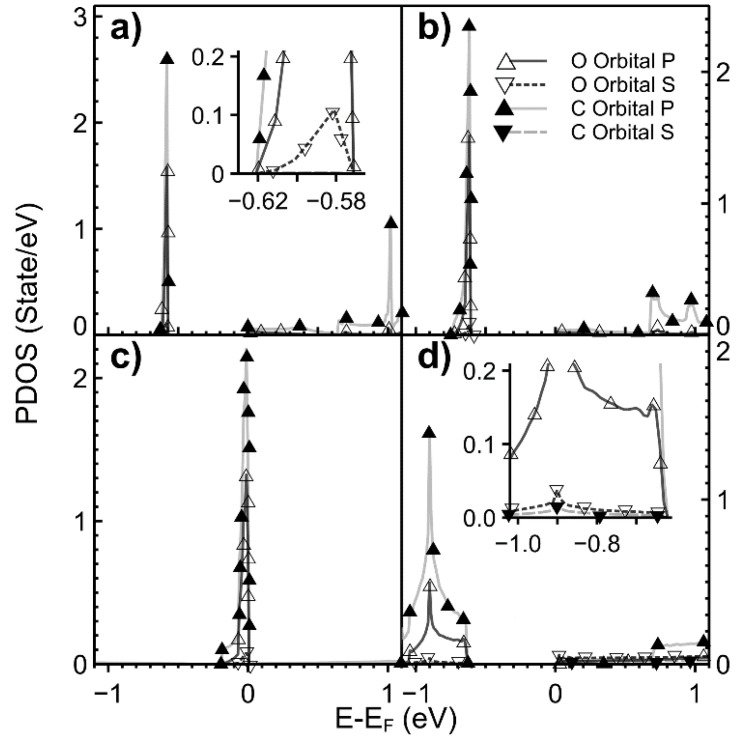
We show the PDOS for different vacancy densities values when the dopant is oxygen. (**a**) 2.00%; (**b**) 3.12%; (**c**) 5.55%; (**d**) 12.5%. For carbon: orbital *s*: ▼ and for orbital *p*: ▲. For oxygen: orbital *s*: ▽; and orbital *p*: △.

In Figure 5, we present the PDOS when the dopant is oxygen. There is a small hybridization of orbitals *s* and *p* around the Fermi energy for carbon in all cases. We can observe a general tendency. The orbitals *s* from carbon and oxygen hybridize with the orbitals *p* from oxygen and carbon in tiny contributions below the Fermi energy. As the dopant density increases, this hybridization occurs at lower points, and the contributions from orbitals s grow. We must mention that this general tendency fails for the densities of 1.39% (not shown in Figure 5) and 5.55%. For these two dopant densities, the hybridization occurs at the Fermi energy.

**Figure 6:**
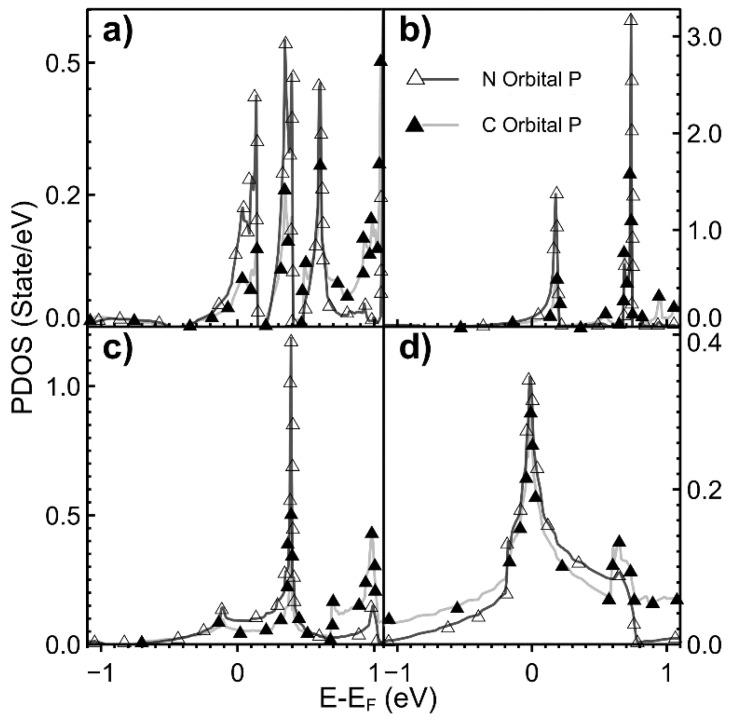
We show the PDOS for different values of vacancy densities when the dopant is nitrogen. (**a**) 2.00%; (**b**) 3.12%; (**c**) 5.55%; (**d**) 12.5%. For carbon; orbital *p*: ▲. For nitrogen: △. The energy range is from −1.0 eV to +1.0 eV. The orbitals s for carbon and nitrogen are too close to zero and don’t appear in the figure.

**Figure 7:**
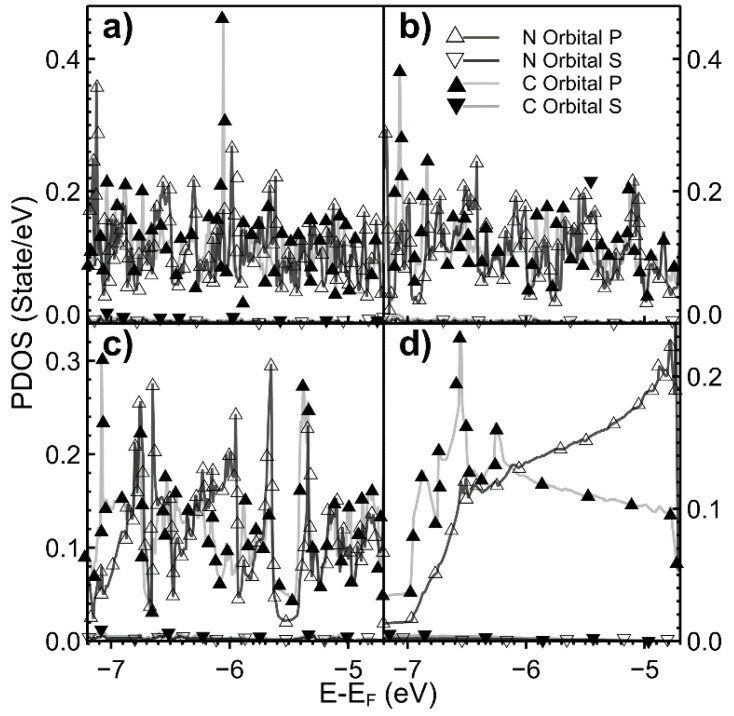
We show the PDOS for different values of vacancy densities when the dopant is nitrogen again. The energy range is from −7.0 eV to −5.0 eV. (**a**) 2.00%; (**b**) 3.12%; (**c**) 5.55%; (**d**) 12.5%. For carbon: orbital *s*: ▼; and for orbital *p*: ▲. For nitrogen: orbital *s*: ▽; and orbital *p*: △. The orbitals *s* for nitrogen and carbon are close to zero.

Figure 6 and Figure 7 show the PDOS when the dopant is nitrogen. For all cases, the hybridization occurs below the Fermi energy (below −5 eV). The contribution of *s* orbitals is tiny. For 0.78% of dopant density (not shown in the figure), this hybridization occurs above the Fermi energy at 2.9 eV.

**Figure 8:**
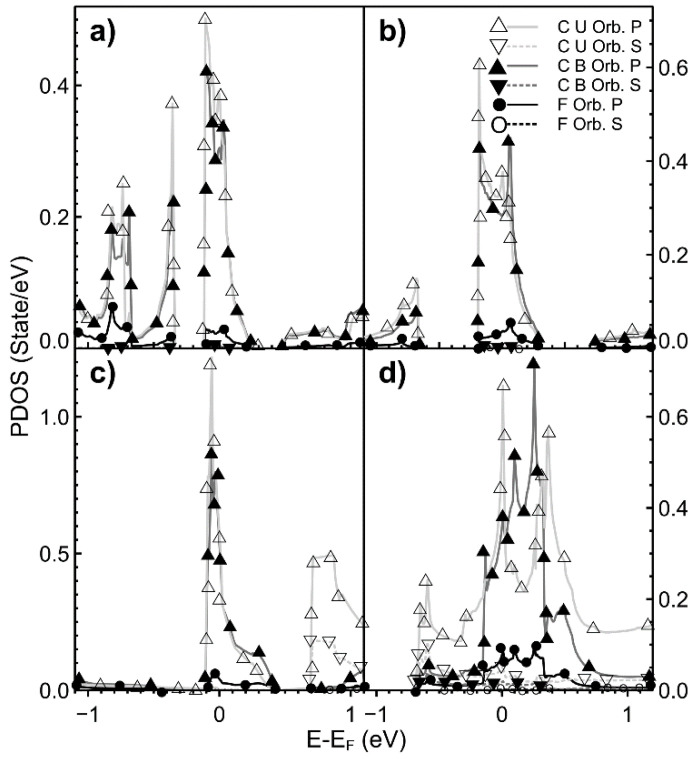
We show the PDOS for different vacancy density values when the dopant is fluorine. In this case, the dopant atom is bound to a carbon atom. (**a**) 2.00%; (**b**) 3.12%; (**c**) 5.55%; (**d**) 12.5%. For unbound carbon: orbital *s*: ▽; and for orbital *p*: △; For bound carbon: orbital *s*: ▼; orbital *p*: ▲. For fluorine: orbital *s*: O; orbital *p*: ●. The energy range is from −1.0 eV to +1.0 eV.

In Figure 8, we show the PDOS for different vacancy density values when the dopant is fluorine. In this case, the contributions of all the *s* orbitals are meager. In all cases, the hybridization occurs at the Fermi energy. We can observe the hybridization of orbitals *s* and *p* above the Fermi energy in all cases. The hybridization is more intense as the density increases. We can see the same kind of hybridization below −0.5 eV.

### 2.3. Electron Transfer

**Figure 9:**
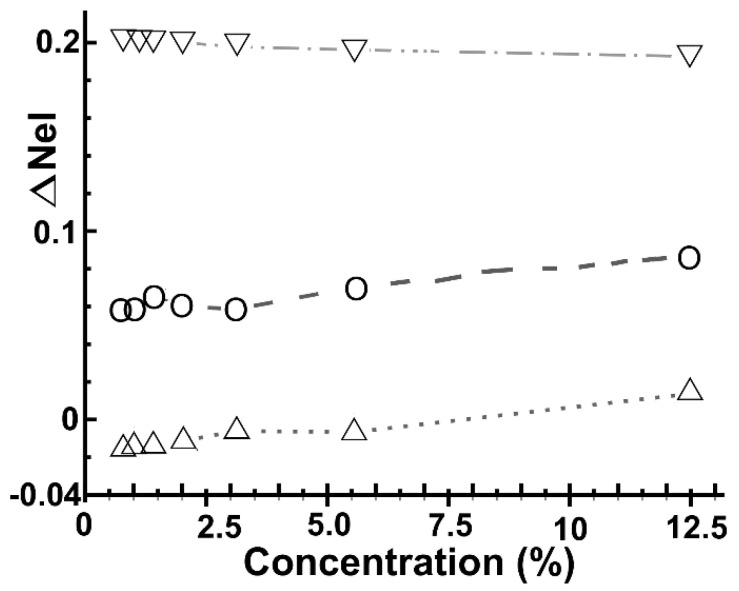
We show the electron transfer for the dopants as a function of their concentration. Nitrogen: △; oxygen: O; Fluorine: ▽.

Figure 9 shows the change in the number of electrons for the dopant atoms with the concentration. In the cases of oxygen and fluorine, the dopants gain electrons. However, nitrogen starts transferring electrons to graphene, and as the dopant concentration increases, the electron loss is minor. When the nitrogen concentration reaches 12.5%, the dopant takes electrons from graphene.

### 2.4. Band Structure

**Figure 10:**
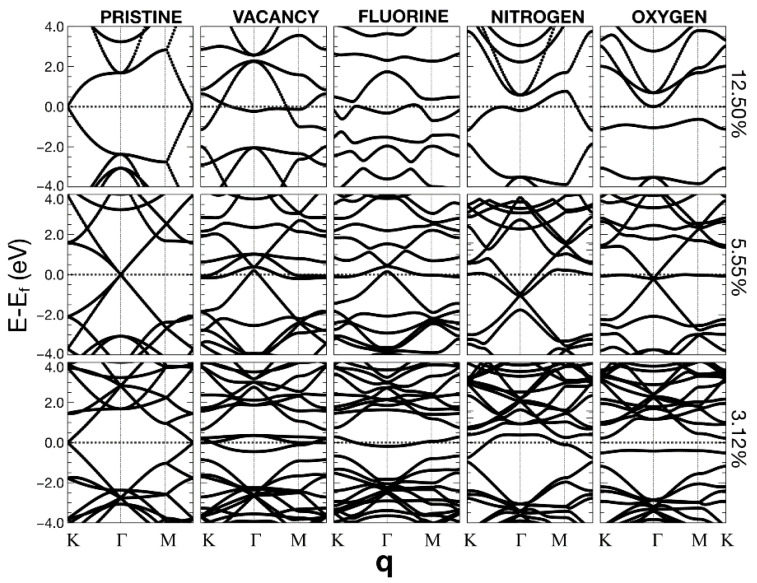
Energy band structures. The left column corresponds to pristine graphene for unit cells of different sizes (containing 2N × N carbon atoms). The 12.5% of dopant (or vacancy) corresponds to a unit cell of 8 bits (N = 2). 5.55% compares to a unit cell with N = 3; 3.12% refers to a unit cell with N = 4. The Fermi energy corresponds to the dotted lines. The second column from the left relates to different vacancy percentages. The middle column shows fluorine’s influence as a dopant on the energy band structure when its presence changes several ratios. The other two columns refer to nitrogen and oxygen, respectively.

**Figure 11:**
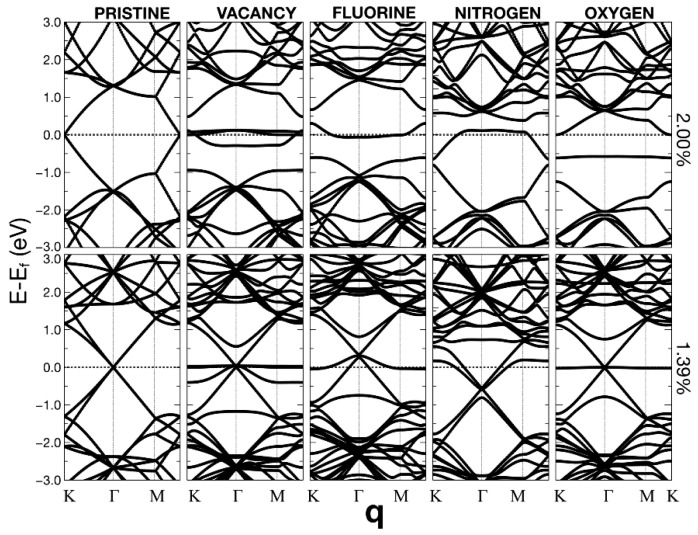
Energy band structures. The left column corresponds to pristine graphene for unit cells of different sizes (containing 2N × N carbon atoms). The 2.0% of dopant (or vacancy) corresponds to a unit cell of 50 bits (N = 5). 1.39% compares to a unit cell with N = 6; The Fermi energy corresponds to the dotted lines. The second column from the left relates to different vacancy percentages. The middle column shows fluorine’s influence as a dopant on the energy band structure when its presence changes several ratios. The other two columns refer to nitrogen and oxygen, respectively.

**Figure 12:**
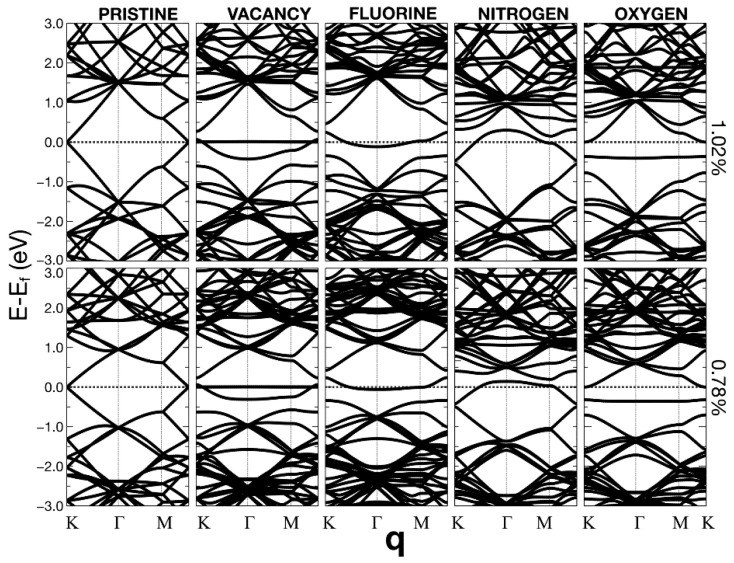
Energy band structures. The left column corresponds to pristine graphene for unit cells of different sizes (containing 2N × N carbon atoms). The 1.02% of dopant (or vacancy) corresponds to a unit cell of 98 bits (N = 7). 1.39% compares to a unit cell with N = 8; The Fermi energy corresponds to the dotted lines. The second column from the left relates to different vacancy percentages. The middle column shows fluorine’s influence as a dopant on the energy band structure when its presence changes several ratios. The other two columns refer to nitrogen and oxygen, respectively.

In Figure 10, Figure 11 and Figure 12, we present the energy band structure for pristine graphene, graphene with a vacancy, and graphene with a dopant for several concentrations.

The surfaces showed some relevant band gap variations. The first column on the left corresponds to pristine graphene for seven different unit cells. Each cell contains 2N × N atoms. The 12.5% of dopant (or vacancy) corresponds to a unit cell of 8 bits (N = 2). 5.55% compares to a unit cell with N = 3; 3.12% refers to a unit cell with N = 4; 2.0% corresponds to 50 atoms (N = 5). 1.39% refers to N = 6; 1.02% refers to a unit cell with N = 7 (98 bits); 1.39% compares to a unit cell with N = 8. Finally, 0.78% corresponds to a unit cell with N = 8 and 128 atoms.

We can observe that the Dirac cones appear at the Κ points, except for the unit cells that contain 18 and 72 atoms (i.e., N = 3 and 6). These unit cells follow the 3N × 3N rule [23]. In these cases, the Dirac point folds from the K to the Γ point. The Brillouin folding is a symmetry consequence that does not affect the description of the system’s properties. Furthermore, and for the same cases, for all the dopants, doped graphene (corresponding to 5.55% and 1.39%) has a zero-gap or a neglectable gap, which occurs in the vacancy case as well. The Dirac points fold from K to Γ points and makes the band diagrams different from the rest. For the other unit cells, there is a gap. The opening of this gap depends on the dopant concentration.

We may say that, in general, the structures are semimetallic. For the vacancies, there is a quasi-flat energy band, around the Fermi energy, for concentrations below 2%. Flat energy bands could imply many-body phenomena (i.e., no-conventional superconductivity or ferromagnetism [24,25,26].

**Figure 13:**
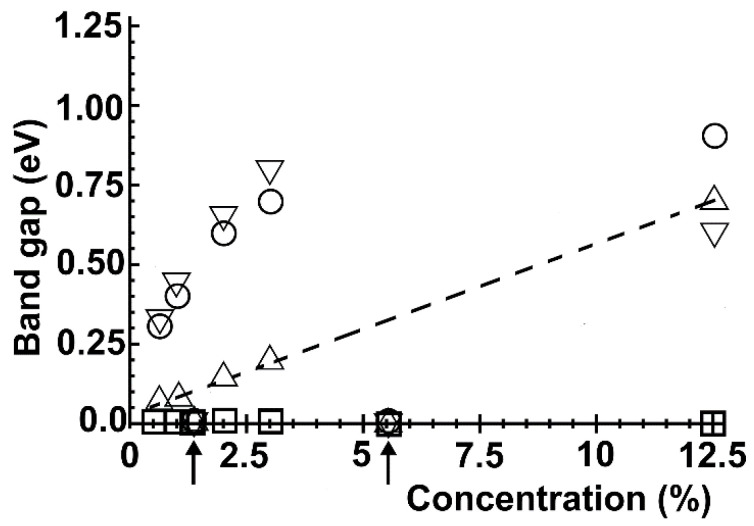
We show the bandgap variation around the Fermi energy for different dopant (vacancy) percentage densities. The arrows indicate the unit cell sizes that follow the 3N rule [23] (1.39% and 5.55%). Nitrogen: △; oxygen: O; Fluorine: ▽; vacancy: □. In the case of nitrogen, there is a linear behavior.

**Figure 14:**
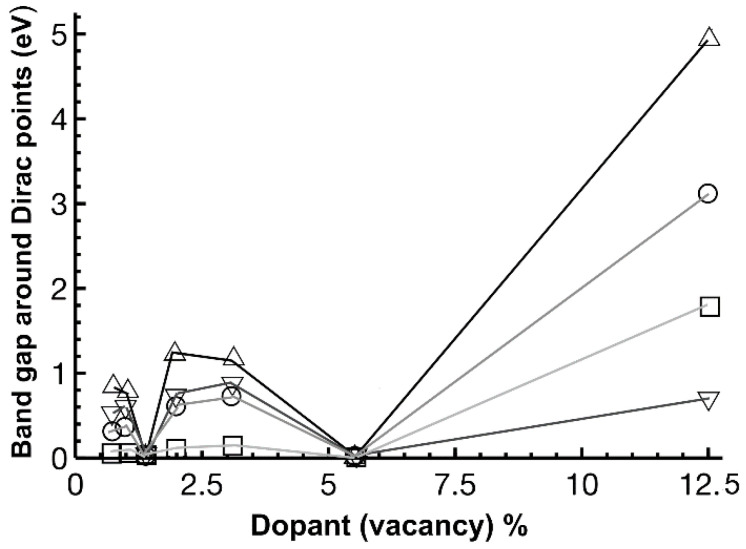
We show the bandgap variation around the Dirac points for different dopant (vacancy) percentage densities. Nitrogen: ▽; oxygen: O; Fluorine: △; vacancy: □. We drew the lines only to keep track of the changes for each dopant. The bandgap collapses to zero for the unit cell sizes that follow the 3N rule [23] (1.39% and 5.55%).

From Figure 13, we see that for each dopant, the most significant gap occurs for the greatest concentration (12.5%, N = 2, i.e., a unit cell with eight atoms). The largest bandgap was for oxygen-graphene (0.95 eV). Furthermore, there is a linear behavior of the bandgap with the dopant concentration in nitrogen-graphene. This behavior agrees with previous reports from other authors [23]. Figure 14 shows the bandgap around the Dirac points. In this case, the biggest bandgap is for fluorine at 12.5%.

### 2.5. Optical Absorption and Reflectivity

**Figure 15:**
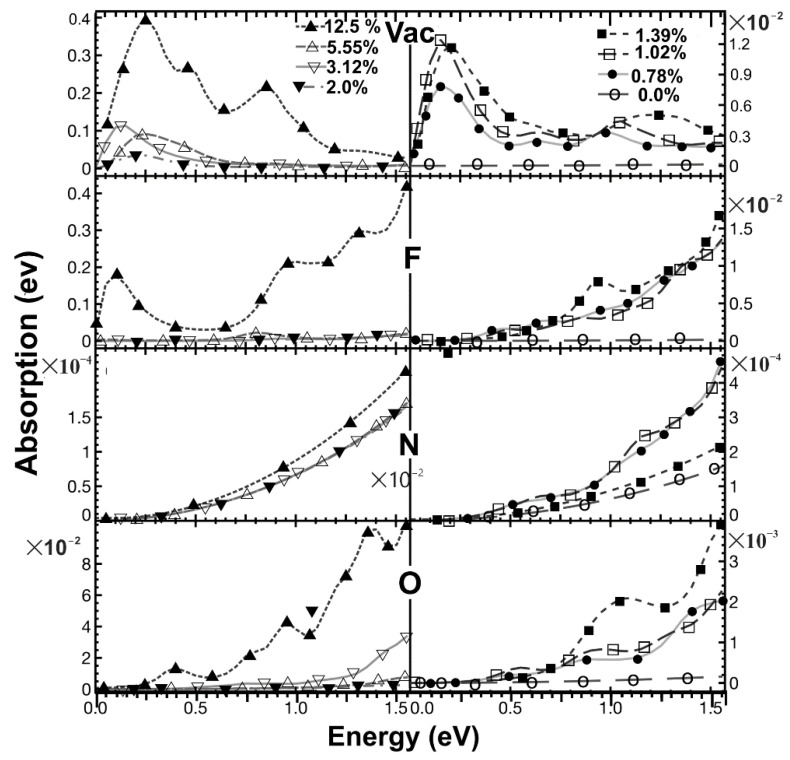
We show the absorption in the infrared region for different dopant (or vacancy) concentrations. For 0% (pristine graphene): Ο; for 0.78%: ●; for 1.02%: □; for 1.39%: ■; For 2.0%: ▼; for 3.12%: ▽; for 5.55%: △; for 12.5%: ▲. At the top, we have the absorption for different vacancy concentrations. At the bottom, we have the optical absorption for different oxygen percentages. As in the two previous cases, we show the absorption for fluorine and nitrogen for the same concentration variation in the middle. The horizontal axis marks the energy of incident electromagnetic radiation.

**Figure 16:**
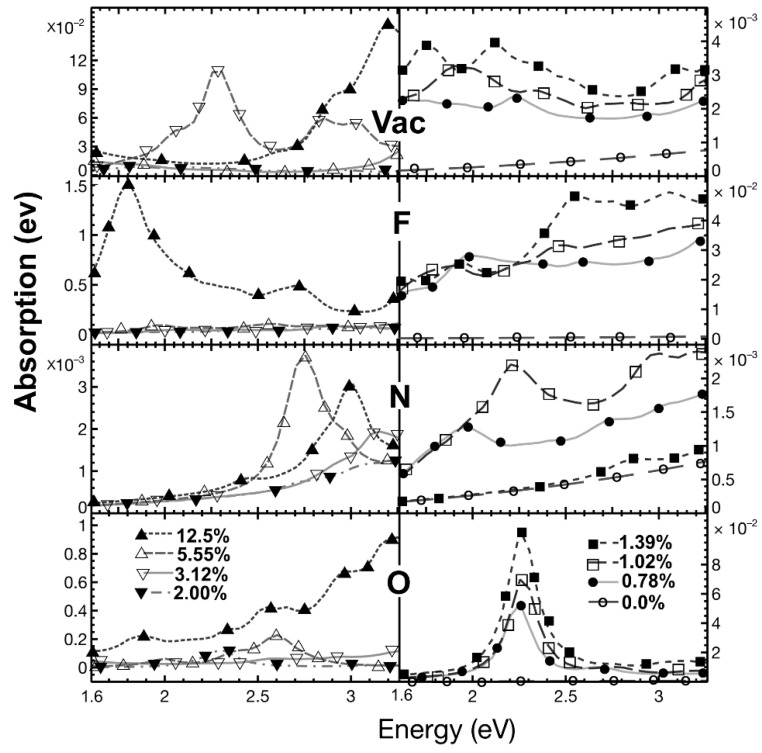
We present the absorption in the visible region for different dopant (or vacancy) concentrations. For 0% (pristine graphene): Ο; for 0.78%: ●; for 1.02% □; for 1.39%: ■; For 2.0%: ▼; for 3.12%: ▽; for 5.55%: △; for 12.5%: ▲. At the top, we have the absorption for different vacancy concentrations. At the bottom, we have the optical absorption for different oxygen percentages. As in the two previous cases, we show the absorption for fluorine and nitrogen for the same concentration variation in the middle. The horizontal axis marks the energy of incident electromagnetic radiation.

**Figure 17:**
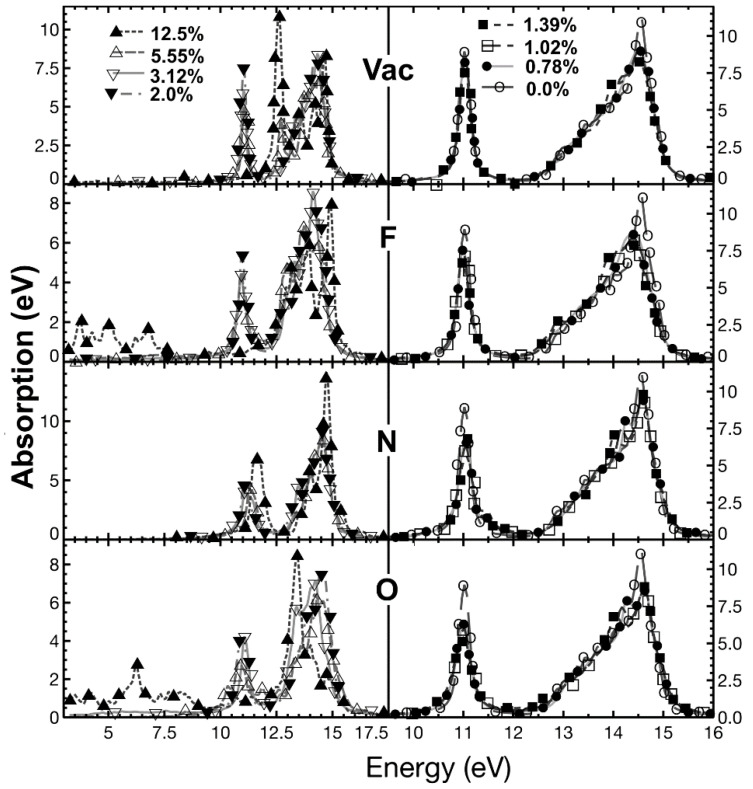
We present the absorption in the ultraviolet region for different dopant (or vacancy) concentrations. For 0% (pristine graphene): Ο; for 0.78%: ●; for 1.02%: □; for 1.39%: ■; For 2.0%: ▼; for 3.12%: ▽; for 5.55%: △; for 12.5%: ▲. At the top, we have the absorption for different vacancy concentrations. At the bottom, we have the optical absorption for different oxygen percentages. As in the two previous cases, we show the absorption for fluorine and nitrogen for the same concentration variation in the middle. The horizontal axis marks the energy of incident electromagnetic radiation.

**Figure 18:**
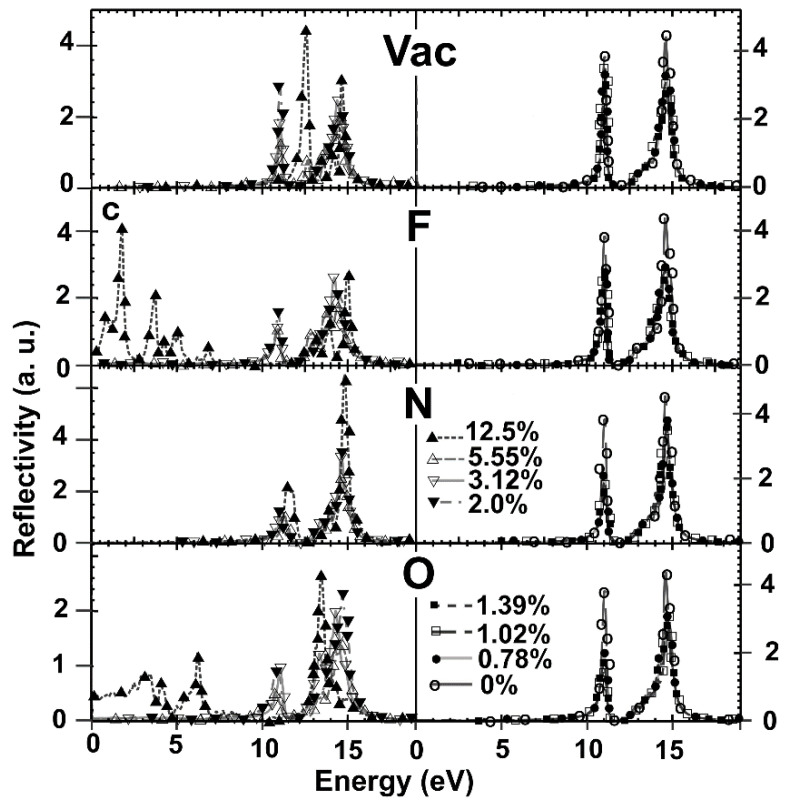
We show the reflectivity for different dopant (or vacancy) concentrations. For 0% (pristine graphene): Ο; for 0.78%: ●; for 1.02%: □; for 1.39%: ■; For 2.0%: ▼; for 3.12%: ▽; for 5.55%: △; for 12.5%: ▲. At the top, we have the reflectivity for different vacancy concentrations. At the bottom, we have the optical reflectivity for different oxygen percentages. As in the two previous cases, we show the reflectivity for fluorine and nitrogen for the same concentration variation in the middle. The horizontal axis marks the energy of incident electromagnetic radiation.

We calculated the dielectric tensor, and we obtained the absorption and reflectivity for each case. We considered the electromagnetic wave propagating in a direction perpendicular to the graphene plane. Our results for pristine graphene agree with reports from other authors [27]. We show our calculations for vacancies and dopants in Figure 15, Figure 16 and Figure 17 for the optical absorption and the reflectivity in Figure 18.

#### 2.5.1. Optical Absorption

In Figure 15, for the infrared region, *pristine graphene* presents minimal absorption. In general, we may say that as the *vacancy* concentration increases, the absorption increments quickly. It is clear that for given incident radiation energy, this increment is not linear. At 12.5% of vacancy concentration, there is a prominent absorption peak at low radiation energies, and then the absorption decreases as the frequency of the incident radiation increments.

When a *fluorine* atom is a dopant, the absorption increases with incident radiation frequency for all concentration values. Furthermore, the absorption increases when concentration increments. For the most significant concentration, there is a local maximum at low frequencies. The absorption has a similar value to the vacancy’s case.

We found that the optical absorption has the same order of magnitude as graphene’s when the *nitrogen* is the dopant. There are no local minima, but there is a slow increment in absorption when the frequency grows.

In *oxygen* as the dopant, the absorption increases as the concentration increments. Furthermore, the absorption has a magnitude more significant than in the nitrogen’s case, but smaller than fluorine’s. The absorption increases when the concentration grows. Overall, we see that the optical absorption increments as the frequency increases.

Figure 16 shows our results for the visible region. We see that *pristine graphene* presents minimal absorption. Again, when the *vacancy* concentration increases, the absorption overgrows. We see a prominent absorption peak for a concentration of 5.55%, which is the maximum absorption at that energy radiation for all concentrations. The location of this peak is around 2.3 eV (i.e., 539 nm, green radiation). There is another maximum for a concentration of 12.5% that occurs at 3.2 eV (i.e., near-ultraviolet region).

With *fluorine* as the dopant, again, the optical absorption increases quickly with concentration. In general, the absorption increases with all concentration values’ energy radiation. However, the maximum absorption occurs for a concentration of 12.5% at the energy around 1.6 eV (i.e., 774.9 nm, red radiation). After that, the absorption decreases with increasing radiation energy. The maximum absorption is closely 1500 times the absorption of graphene.

In *nitrogen* as the dopant, the optical absorption magnitude is two or three times larger than the graphene’s corresponding absorption. The two significant peaks happen for concentrations of 5.55% and 12.5%. The first one occurs around 2.75 eV (i.e., 450 nm, blue radiation), and the second at about 3 eV (i.e., 413 nm, violet radiation).

When *oxygen* is the dopant, for concentrations below 2%, there is a peak value for the optical absorption. This peak occurs at 2.25 eV (i.e., 551 nm, green radiation). Furthermore, in general, the absorption increase as the dopant concentration increments. When the dopant percentage is 12.5%, the absorption grows with frequency. In this case, the absorption is around one thousand times the corresponding absorption of graphene.

In Figure 17, we present the results for the optical absorption for the ultraviolet region. In this range, *pristine graphene* shows two prominent peaks. One is for incident radiation of 11.02 eV, and the other for 14.58 eV. The absorption resembles pristine graphene for vacancy concentrations below 5.55%*;* when the percentages are 5.55% and 12.5%, the maximum on the left moves about 1.5 eV to the right.

When *fluorine* is the dopant, the absorption, again, resembles pristine graphene for dopant percentages below 5.55%, for concentrations 5.55% and 12.5%, the maximum on the left moves to the right about 2.5 eV. In this way, the two maxima come close to each other. Furthermore, for 12.5% of dopant concentration, three small peaks appear below 7.5 eV.

In the *nitrogen* case, the changes are tiny. The most noticeable is 12.5% concentration, where the peak on the left moves about 1 eV to the right.

When *oxygen* is the dopant, there are few changes concerning pristine graphene for all dopant concentrations, except for 12.5%. There is a tendency to move the peak on the right slightly to the left as the percentage increases. However, for 12.5%, the maximum on the right moves about 1.5 eV to the left. Furthermore, the peak on the left moves about 5.0 eV to lower energies.

#### 2.5.2. Reflectivity

In Figure 18, we present the reflectivity for the whole range of energy of incident radiation. The reflectivity of *pristine graphene* shows two maxima located in the ultraviolet range. One is for radiation of 11.07 eV, and the other for 14.57 eV. For *vacancies*, we see that for all concentrations, the reflectivity resembles pristine graphene. There is only a noticeable change at 12.5%; the peak on the left moves 0.3 eV to the right, getting closer to the other maximum.

When *fluorine* is the dopant for all concentrations, the reflectivity resembles graphene. There is a significant change for 12.5%. In this case, the peak on the right moves 0.1 eV to the right. Furthermore, the maximum on the left disappears, and five peaks appear below 8 eV.

In the case of *nitrogen*, the reflectivity is similar to that of graphene for all dopant concentrations. There are tiny changes only when the percentage is 12.5%. In this case, the two peaks move to the right about the same amount, 0.12 eV.

Finally, with *oxygen* as a dopant happens something similar to fluorine. In general, the reflectivity resembles graphene for all concentrations except at 5.55% and 12.5%. In the case of 5.55%, one additional maximum appears between the two prominent peaks. At 12.5%, the maximum on the right moves 0.15 eV to the left. The peak on the left disappears, and three maxima appear below 8 eV.

## 3. Discussion

We investigated the changes in the structure of the energy bands and the optical properties of graphene when the concentration of vacancies and doping changed. We considered fluorine, nitrogen, and oxygen as dopants. We investigated the concentration values 0.78%, 1.02%, 1.39%, 2.00%, 3.12%, 5.55%, and 12.5%.

The three dopant atoms chemisorb in the graphene surface, with absorption energies that suggest stability of the resulting systems. In the three cases, the magnitude of the adsorption energies decreases with increasing doping density almost linearly. We found that fluorine showed the lowest adsorption energy and nitrogen the highest. Nonetheless, we found that the dopant density influences the hybridization of the atomic orbitals and the energy band structure. Oxygen as a dopant at a concentration of 12.5% presents the largest bandgap (0.95 eV). Fluorine shows a maximum bandgap of 0.8 eV at a concentration of 3.12%. The widest bandgap we obtained for nitrogen as a dopant was 0.70 eV at a concentration of 12.5%.

We found substantial variations in the optical properties when the vacancy and doping concentrations change. The most significant changes in reflectivity are with fluorine and oxygen as dopants. Five maxima appear below 8 eV, where the pristine graphene shows a near-zero reflectivity in fluorine at a concentration of 12.5%. The highest of these maxima has an intensity of 20% of the maximum reflectivity in pristine graphene. One of the two prominent peaks in graphene disappears. Something similar happens with oxygen: three peaks appear below 8 eV at 12.5%. The most intense of these peaks is 22% of the highest maximum in pristine graphene. One of the two prominent peaks in pristine graphene also disappears.

For the optical absorption, there are only two maxima in pristine graphene and occur in the ultraviolet range. In the rest of the spectrum, the absorption is near zero. In the infrared, in all cases (including vacancies), we obtained significant changes concerning pristine graphene. Fluorine, at a concentration of 12.5%, shows the most significant change. The most prominent peak was 4.0% of the maximum absorption in pristine graphene. We should mention that the second-largest absorption occurred for the vacancies and was 3.6% of the most prominent peak in graphene.

In the visible range, something similar happens. For all cases, vacancies included, there is a substantial increment in the absorption. The most significant changes are for fluorine and oxygen as dopants at a concentration of 12.5%. The maximum absorption for fluorine was 13.6% of the most considerable absorption in graphene and for oxygen was 8.2%.

For the ultraviolet region, the most relevant changes concerning pristine graphene occur at a concentration of 12.5% for all instances, including vacancies. However, for oxygen and fluorine, the peak on the left disappears. In oxygen, three maxima appear below 8 eV. The maximum of these three is 27.2% of the maximum absorption in graphene. In fluorine, five maxima appear below 8 eV, and the most prominent of these reaches 22.7% of the maximum peak for pristine graphene.

We found significant variations in the optical properties, especially in the infrared and visible ranges, implying changes in the material’s color. The changes in the tincture of defected or doped graphene may be a valuable tool for detecting the concentration of dopants or vacancies.

## 4. Materials and Methods

We utilized density functional theory (DFT) via the Quantum ESPRESSO code [28,29]⁠ to perform all the calculations under the pseudopotential and plane wave approximation. We considered an energy cut-off value of 1400 eV, the generalized gradient approximation (GGA), and the Perdew–Burke–Ernzerhof (PBE) expression for the exchange-correlation functional [30]. We calculated the adsorption energy, the energy band structure, the projected density of states (PDOS), and the dielectric tensor for each case. For this purpose, we utilized norm-conserving pseudopotentials with the Goedecker–Hartwigsen–Hutter–Teter method and the Van der Waals VdW-DFT [31,32,33,34] correction. We took a 8 × 8 × 1 k-point mesh within the Monkhorst–Pack scheme [35]. The energy convergence threshold was 10^−6^ eV. We considered a supercell size large enough to avoid spurious interactions. In this way, the cell size on the z-axis was 15 Å.

Many studies are focused on calculating the properties when the dopant is inside the graphene structure. We perform a geometrical optimization with the dopant out of graphene’s plane to analyze which kind of interaction would be natural. Thus, we placed each dopant atom at 3 Å from the vacancy graphene sheet. In all cases, we optimized the graphene surface with vacancies. We then added the dopant and performed a geometry optimization. We calculated the adsorption energies using the formula (1):

(1) ΔE=E(final structure)−[E(X)+E(vacancy−graphene)] 

where *X* = N, O, F.

For visualizations, we used the XCrySDen software [36]. We calculated the corresponding band structures, densities, and projected densities of states (PDOS). We obtained the optical properties, absorption, and reflectivity from the complex dielectric function. We obtained the imaginary component of the dielectric tensor from electronic bands’ structure in the linear optics limit. Within the random phase approximation (RPA) [37],

(2) $$\varepsilon_{\alpha \beta }\left(\omega \right)=\frac{4n^{2}}{m^{2}\omega^{2}}\underset{c,\upsilon }{\sum }\int d^{3}k\langle c_{k}|p^{\alpha }|\upsilon_{k}\rangle \langle \upsilon_{k}|p^{\beta }|c_{k}\rangle \delta \left(\varepsilon_{ck}-\varepsilon_{\upsilon k}-\omega \right)f_{c}\left(1-f_{\upsilon }\right)$$

In Equation (2), *n* is the electron density, *m* is the effective mass, and *ω* is the photon frequency. The indices *α* and *β* are the cartesian components of *p* that is the vector defining the incident electric field’s polarization. The symbols *c_k_* and *υ_k_* are the wave functions corresponding to the conduction, and the valence bands with crystal wave vector *k*; *f_c_* is the Fermi distribution function for the state *c*. The sum of the transitions from occupied to unoccupied states is over the first Brillouin zone. Finally, we weigh the probability of a change.

By the Kramers-Kronig relations [38] and using Equation (2)

(3) Reεαβ(ω)=δαβ+2nP∫0∞ω′Imεαβ(ω)ω′2−ω2dω′

Considering a perpendicular incidence of the electromagnetic wave, the reflectivity and absorption *Rii* and *Aii* are (with *n* and *k* being the refractive index and extinction coefficient, respectively):

(4) $$R_{ii}(\omega )=\frac{{\left(n-1\right)}^{2}+k^{2}}{{\left(n+1\right)}^{2}+k^{2}}$$

(5) $$A_{ii}\left(\omega \right)=\frac{2\omega k\left(\omega \right)}{c}$$

And

(6) nii=|εii(ω)|+Reεii(ω)2

(7) kii(ω)=|εii(ω)|−Reεii(ω)2

## Figures and Tables

**Table 1:** Absorption energies (in eV) for each of the 21 cases of substitutional doping we considered after performing the structural relaxations.

% Doping	F-Graphene	N-Graphene	O-Graphene	Atoms in the Unit Cell
12.5	−4.373371287	−12.83995707	−7.955791861	8
5.55	−4.806661273	−13.78657355	−8.806335029	18
3.12	−5.137406876	−13.99255507	−8.953832994	32
2.00	−5.266265993	−14.01279697	−8.967541945	50
1.39	−5.323285282	−14.02436461	−8.939755112	72
1.02	−5.403380587	−14.03414828	−8.964265388	98
0.78	−5.439137917	−14.04038286	−8.967093484	128
